# Nonsurgical Management of an Immature Maxillary Central Incisor with Type III Dens Invaginatus Using MTA Plug: A Case Report 

**DOI:** 10.22037/iej.v12i4.17769

**Published:** 2017

**Authors:** Negar Norouzi, Majid Kazem, Atefeh Gohari

**Affiliations:** a *Department of Endodontics, Dental School, Mazandaran University of Medical Sciences, Mazandaran, Iran; *; b *Department of Endodontics, Dental School, Shahid Beheshti University of Medical Sciences, Tehran, Iran*

**Keywords:** Dens Invaginatus, Maxillary Central Incisors, MTA Plug, Non-Surgical Endodontic Treatment

## Abstract

Dens invaginatus is a developmental anomaly, caused by deepening of the enamel organ into the dental papilla before calcification of the dental tissues. Teeth with dens invagination are susceptible to early caries and pulp necrosis within a few years of eruption or even before root end closure. This article reports two immature maxillary central incisors with type I and III dens invaginatus which had necrotic pulp and a large periradicular lesion, that were treated successfully by nonsurgical root canal treatment. After apical plug placement, the remaining space was backfilled using warm vertical gutta-percha technique and the crowns were restored by composite restoration. At 6 months of follow up the patient was asymptomatic and probing depths were less than 3 mm. In addition, the reduction in the size of apical radiolucencies was observed by radiographic examinations. This case report revealed that even type III des invaginatus with an open apex and large periapical lesion, can be treated non-surgically using MTA as an apical plug. Although this case report presents a favorable result, further studies with long term follow-up periods are encouraged to support the use of nonsurgical endodontic treatment for type III dens invaginatus.

## Introduction

Dens invaginatus is a developmental variation resulting from a modification in the normal growth of the dental papilla [1]. It is caused by deepening or invagination of the enamel organ into the dental papilla before calcification of the dental tissues [2]. It is also known as Dens in dente, invaginated odontome, dilated gestant odontome, dilated composite odontome, tooth inclusion and dentoid in dente [2]. This variation in nomenclature probably reflects lack of agreement on the formation, etiology and classification of this condition [3]. The prevalence of adult teeth affected with dens invaginatus is between 0.3% and 10% [4]. Dens invagination could be present in both deciduous and permanent dentition [5] and both maxillary and mandibular arches [1], but the permanent maxillary lateral incisor seems to be the most frequently affected tooth [2] with posterior teeth less likely to be affected [6]. In addition, dens invaginatus is more frequent in men, by a ratio of 3:1 [7]. 

The etiology of dens invaginatus is controversial. Most authors consider dens invaginatus as an infolding of enamel and dentin beginning from the foramen coecum during tooth development [2, 8]. Other factors include failure of growth of a part of the internal enamel epithelium while the surrounding normal epithelium continues to proliferate [9], rapid and aggressive proliferation of a part of the internal enamel epithelium invading the dental papilla [10], distortion of the enamel organ during tooth development [11], fusion of two tooth-germs, infection [12], trauma [13] and genetic factors [14-17].

**Figure 1:**
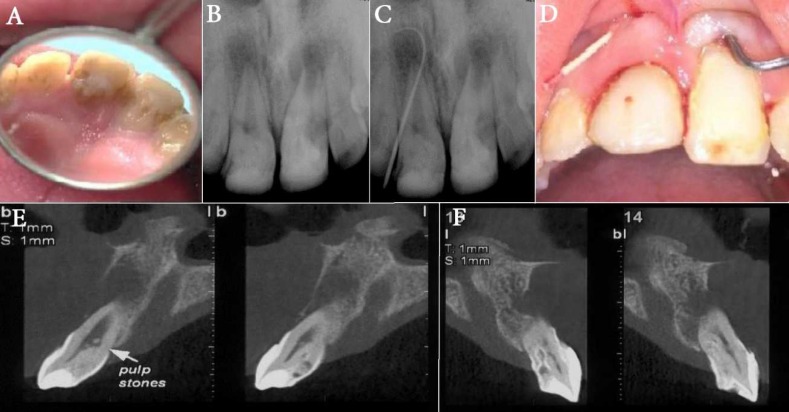
*A)* Preoperative anatomic variation of the palatal aspect of the crown revealed on the clinical examination*;* *B)* Preoperative periapical radiograph, shows the origin of the sinus tract*;* *C)* Preoperative radiograph, shows the origin of the sinus tract*;* *D)* A deep narrow pocket was observed on midbuccal sulcus of tooth #9. Its pathologic migration is also obvious on this photograph*;* *E and F)* CBCT image of teeth #8 and #9 showed type I dens invaginatus in tooth #8 and type III dens invaginatus in tooth #9, based on Oehlers’ classification

The most commonly used classification is suggested by Oehlers who classified dens invaginatus into three types based on the severity of the defect. Type 1 dens invaginatus is an invagination restricted to the crown. Type 2 extends past the cemento enamel junction (CEJ) but does not involve periapical tissues. The most severe and the most complex form to treat is the Type 3 defect. The invagination extends past the CEJ and may result in a second apical foramen [11].

Treatment of teeth with dens invaginatus ranges from prophylactic restorative procedures (if diagnosed early) to nonsurgical root canal therapy, surgery, or even extraction. Dentists faced by dens invaginatus associated with large periapical lesions encounter difficulties in treating these teeth.

This article reports and discusses two immature central incisors with dens invaginatus type I and III which had necrotic pulp and a large periradicular lesion, were treated successfully by nonsurgical root canal treatment.

## Case Report

A 14 year-old female patient was referred to the Department of Endodontics at the School of Dentistry, Shahid Beheshti University of Medical Sciences (Tehran, Iran) for evaluation. She reported periodic pus drainage from buccal gingiva of the maxillary centrals as her chief complaint. Her medical History was non-contributory. Extra-oral examinations showed no facial asymmetry, no swelling and cervical and sub-mandibular lymph nodes were normal on palpation. Intra-oral examinations revealed anatomic variation of teeth #8 and #9 with a sinus tract in the buccal alveolar mucosa, adjacent to the tooth #8 (Figure 1A). In order to trace the sinus tract, a size #25 gutta-percha cone (Meta Biomed Co. Ltd, Cheongju, Korea) was threaded into the opening of the sinus tract until resistance was felt. After a periapical radiograph was exposed (Figure 1B), the origin of the sinus tract was located apical to the tooth #8. There was gingival recession and pathologic migration related to tooth #9. A narrow deep pocket was probed at mid buccal of tooth #9 (Figure 1C). Both teeth #8 and #9 had no response to cold (DENRONIC, Aeronova GmbH & Co. KG, Germany) and electric pulp testing (Elements Diagnostic Unit, Sybronendo, Redmond, WA, USA), grade II mobility and no sensitivity to percussion and palpation. Lateral incisors were also tested and responded normal to thermal and EPT tests with no sensitivity to percussion or palpation.

Radiographic examinations revealed the presence of Dens Invagination in both central incisors (Figure 1D). It was categorized as type I dens invagination for tooth #8 and type III for tooth #9 based on Oehlers classification. An extended radiolucent area in the apical region of #8 and #9 teeth was also noted. The patient ordered to take a CBCT that confirmed previous categorization of the present dens invaginations (Figure 1E). It also showed that the large periapical radiolucency has perforated the buccal cortical bone.

**Figure 2:**
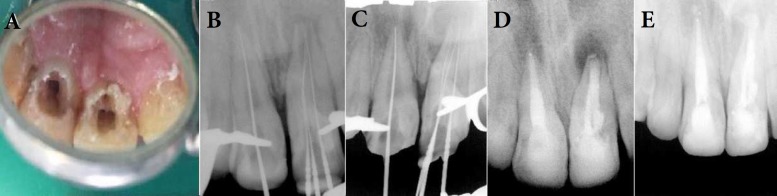
*)* Prepared access cavities. Note that three orifices are detectable on tooth #9; *B, C)* Working length determination; *D)* Post treatment radiograph. Obturation was performed using MTA plug (6 mm of MTA) as apical barrier and backfilling with warm vertical technique; *E)* Follow up radiographs showed significant reduction in the size of apical radiolucencies on 3 months after treatment

Based on subjective, objective and radiographic findings, diagnosis was made as type I and III dens invagination for teeth #8 and #9, respectively, pulp necrosis and chronic apical abscess. It was diagnosed that a primary endodontic lesion led to a secondary periodontal involvement. Hence, nonsurgical endodontic treatment with calcium hydroxide therapy, following by composite restoration and follow up was planned. The possibility of surgical intervention should be mentioned and the patient should be referred for periodontal management.

Treatment plan was explained to the patient and her parents. Written informed consent was obtained. At first appointment, under local anesthesia through infiltration of 2% Lidocaine with 1:80000 epinephrine (Persocaine-E, Darou Pakhsh Pharmaceutical Mfg Co., Tehran, Iran) and rubber dam isolation (Split dam), access cavities were prepared for both central incisors using a diamond fissure bur (Jota AG, Rüthi, Switzerland) (Figure 1F). Pus was allowed to drain, so that drainage of the abscesses was performed through an intra-canal path. The presence of necrotic pulp tissue and pus drainage confirmed the initial diagnosis. The working length of both canals was determined using an electronic apex locator (Root ZX apex locator, J. Morita USA, Inc., Irvine, CA, USA) and confirmed radiographically (Figure 2A). Chemo-mechanical cleaning and shaping was performed using NiTi rotary files (ProTaper Universal, Dentsply Maillefer, Ballaigues, Switzerland) and conventional stainless steel hand K-files. Disinfection was accomplished irrigating with 2.5% sodium hypochlorite alternated with normal saline. Also 17% EDTA was used as the final irrigant in order to remove the smear layer. The canals were dried to some extent using absorbent paper points (Aria Dent, Tehran, Iran). Subsequently, a creamy paste of calcium hydroxide (CH) (Merck, Darmstadt, Germany) was placed as intra-canal medication and the tooth was restored temporarily with Coltosol (Aria Dent, Tehran, Iran).

At second appointment, which was set for 2 weeks after first appointment, clinical examinations showed grade I mobility; therefore, an improvement in periodontal condition was assumed; however, intra-canal exudation and persistent sinus tract do not let the obturation begin. Consequently, previous CH was replaced with a dense paste of CH in both canals for 4 weeks.

At third appointment, CH dressing was irrigated with 2.5% sodium hypochlorite alternated with 17% EDTA and a final flush of normal saline. Drainage had been stopped and completely dried canals were achievable. Both canals were dried relatively using paper points. The root canal spaces were obturated using OrthoMTA (bioMTA, Seoul, Republic of Korea) plug as an apical barrier and back filled with warm vertical compaction technique using gutta-percha (Meta Biomed Co. Ltd, Cheongju, Korea) and root canal sealer (AH-26, DeTrey, Dentsply, Konstanz, Germany) (Figure 2B). Access cavities of both teeth were temporarily sealed with Coltosol (Aria Dent, Tehran, Iran) and the crown was restored permanently with composite restoration one week later.

Additionally, in consultation with periodontal department, patient was instructed to use Zero Listerine mouthwash and Chlorhexidine gel because of her poor oral hygiene which resulted in gingivitis. Tooth #9 was pathologically migrated, hence needs occlusal adjustment. Connective Tissue Grafting (CTG) and leveling of gingiva margins was also suggested to the patient, because of insufficient keratinized gingiva and unleveled gingival margins. 

At 6 months of follow up, both teeth were asymptomatic and clinical examination revealed no swelling, no sensitivity to percussion or palpation. Probing depths were less than 3 mm and the mobility was within normal limits. In addition, the reduction in the size of apical radiolucencies was observed by radiographic examinations (Figure 2C).

## Discussion

In teeth with dens invaginatus, the invagination allows entry of irritants into a space which is either separated from pulpal tissue by only a thin layer of enamel and dentine [2, 18], or some channels may facilitate communication between the invagination and the pulp tissue [9, 19]. Thus, these teeth are susceptible to early caries and pulp necrosis. Pulp necrosis usually occurs within a few years of eruption, sometimes even before root end closure [20-25]. Undiagnosed dens invaginatus may result in abscess formation, retention of neighboring teeth, displacement of teeth, cysts [6] and internal resorption [2]. 

Early diagnosis of dens invagination is mandatory for starting preventive treatment [26]. In most cases a dens invaginatus is detected by chance on the radiograph. Clinically, an abnormal crown shape (dilated, peg-shaped or barrel-shaped) or a deep foramen coecum may be important hints, but affected teeth also may reveal no clinical signs of the malformation [2]. According to the Joint Position Statement of the American Association of Endodontists and the American Academy of Oral and Maxillofacial Radiology on the Use of cone-beam computed tomography (CBCT) in Endodontics [27], limited field of view should be considered as the imaging modality of choice for initial treatment of teeth with dental anomalies. 

Treatment of teeth with dens invaginatus ranges from preventive restorative procedures (if diagnosed early) to nonsurgical root canal therapy, surgery, or even extraction.

Different treatment options have been suggested for these teeth, depending on the severity of the infection and the degree of complexity of tooth anatomy. If there is no entrance to the invagination and no sign or symptoms of pathosis can be detected on clinical and radiographic examinations, no treatment is indicated; although strict observation is still recommended [28, 29]. Teeth with deep palatal, incisal invaginations or foramina coeca should be sealed with fissure sealant materials as a preventive approach. In these cases, composite restoration and strict periodic review is again recommended [30]. In case of pulp exposures as a result of caries, trauma or mechanical tooth preparation and in teeth exhibiting provoked pain of short duration, relieved by over-the-counter analgesics, without signs and symptoms of irreversible pulpitis, which have a clinical diagnosis of reversible pulpitis and are candidates for vital pulp therapy, partial pulpotomy is the preferred option in elective treatment procedures for teeth with dens invaginatus [31]. The treatment plan for necrotic pulp cases can be conservative nonsurgical root canal treatment either associated or not with endodontic apical surgery, intentional replantation and extraction [32-34]. A combination of nonsurgical and surgical therapy is often used to successfully treat type 2 and type 3 cases [35-41].

Nonsurgical root canal treatment should be attempted as the first step. Surgical treatment is the second option and is only indicated in case of failure of the conservative root canal treatment, in teeth which cannot be treated nonsurgically because of anatomical variations or failure to gain access to all parts of the root canal system [30, 32, 42-45]. Intentional replantation is also suggested in hopeless cases [46, 47]. Extraction is only indicated when unusual crown morphology led to aesthetic or functional problems, in teeth which cannot be treated non-surgically or by apical surgery and in supernumerary teeth [30].

Root canal treatment may be difficult and the clinician may encounter several problems because of the irregular, circular or narrow shape of the root canal system [48]. The large and irregular volume of the root canal system makes proper cleaning and shaping difficult. Therefore, irrigation by means of ultrasonic activation has been suggested as an efficient method of disinfection [49, 50]. In addition, the use of intra-canal medications such as CH which had antimicrobial action and tissue dissolving effect [51] is necessary. In this case, in order to optimize cleaning of the canal, placement of CH was considered. At the time of treatment, we had no access to ultrasonic devices; instead we used manual dynamic irrigation. Manual activation was done with a tapered master gutta-percha point [52]. 

When pulp necrosis occurs before root-end closure, apexification procedures with long-term CH or Mineral Trioxide Aggregation (MTA) plug is necessary [20, 21, 53-56]. Use of collagen membranes has also been suggested to provide apical barrier against which MTA cement could be packed [57]. In this case MTA was used as an apical barrier, because the treatment could be completed in one appointment; so improved patient compliance can be achieved. MTA barrier also has the advantages including reduced cost of clinical time and the ability to securely restore the tooth at an earlier stage [58]. The risk of tooth fracture due to long-term use of CH medication is also eliminated [59]. In the present case, obturation was delayed until setting of the MTA had been completed, because MTA has a slow setting reaction.

In order to obdurate teeth with dens invaginatus, warm gutta-percha techniques including vertical condensation or thermoplastic filling techniques have been recommended [30, 60]. In the present case, right after apical closure, a warm vertical technique obturation was performed. Warm gutta-percha can flow into irregularities within the canal system and push sealer into minor irregularities.

## Conclusion

This case report revealed that even type III des invaginatus with an open apex and large periapical lesion, can be treated non-surgically using MTA as an apical plug. Although this case report presents a favorable result, further studies with long term follow-up periods are encouraged to support the use of nonsurgical endodontic treatment for type III dens invaginatus.
